# Supplementary material for: Lights, Camera, Interaction: Studying Protein–Protein Interactions of the ER Protein Translocase in Living Cells
Source: Int J Mol Sci. 2021 Sep 26;22(19):10358. doi: 10.3390/ijms221910358 (PMC8508666; doi:10.3390/ijms221910358)
Supplement: Supplementary file 1 [file ijms-22-10358-s001.zip › ijms-1347448-supplementary.pdf]

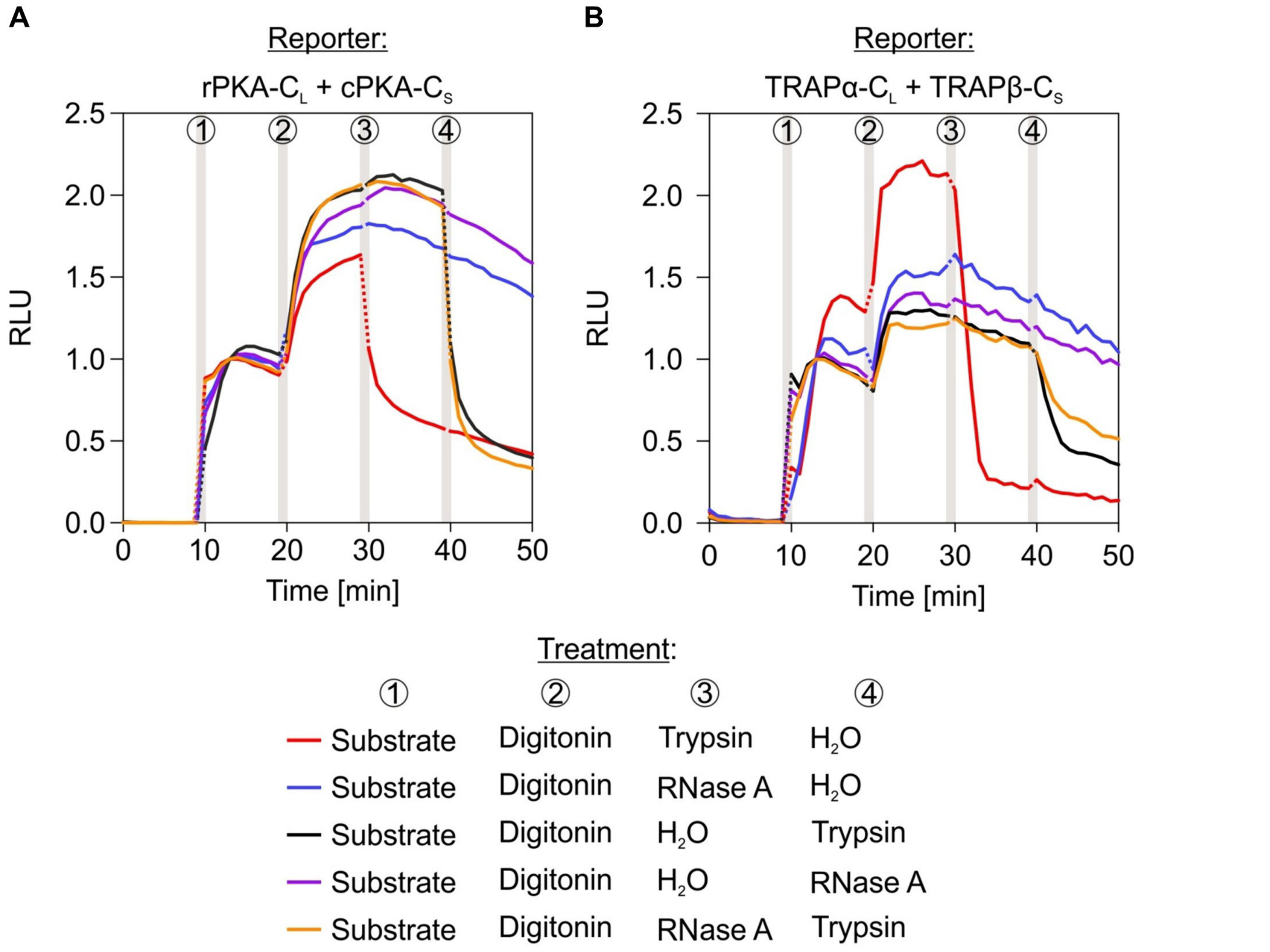

Figure S1. Elimination of luminescence by trypsin-mediated reporter degradation
(**A**) 24 h post transfection with the rPKA-C_L and cPKA-C_S reporter pair cells were subjected to the indicated treatment regimens (circled numbers). After the stimulation of luminescence by furimazine and the permeabilization of the plasma membrane by digitonin (0.002 %), the cells were treated either with the non-proteolytic RNase A (80 µg/ml) or the protease trypsin (50 µg/ml) to digest the reporter and other cytosolic proteins. A water treatment served as vehicle control for RNase A and trypsin, respectively. The DMSO treatment as vehicle control for digitonin is shown in Fig. 1D. Measurements were normalized to the signal intensity recorded 4 min after furimazine application. (**B**) Same as in (**A**), but cells were transfected with the TRAPα-C_L and TRAPβ-C_S reporter pair representing two membrane proteins of the ER (cf. Fig. 6A, B). Vertical grey bars in the line diagrams represent manual 1 min application periods without luminescence readings. The dotted lines are extrapolated based on the last and first data points before and after application. C_L, C-terminally located LgBiT tag; C_S, C-terminally located SmBiT tag

A

Peptide	Sequence	Purpose
Actin	DDDIAALVVDNGSGMC	Negative control
HaBiT	VSGWRLFKKIS	High affinity LgBiT interactor
Scrambled	ERGTCLELYVIF	Negative control
SmBiT	CVTGYRLFEEIL	Low affinity LgBiT interactor

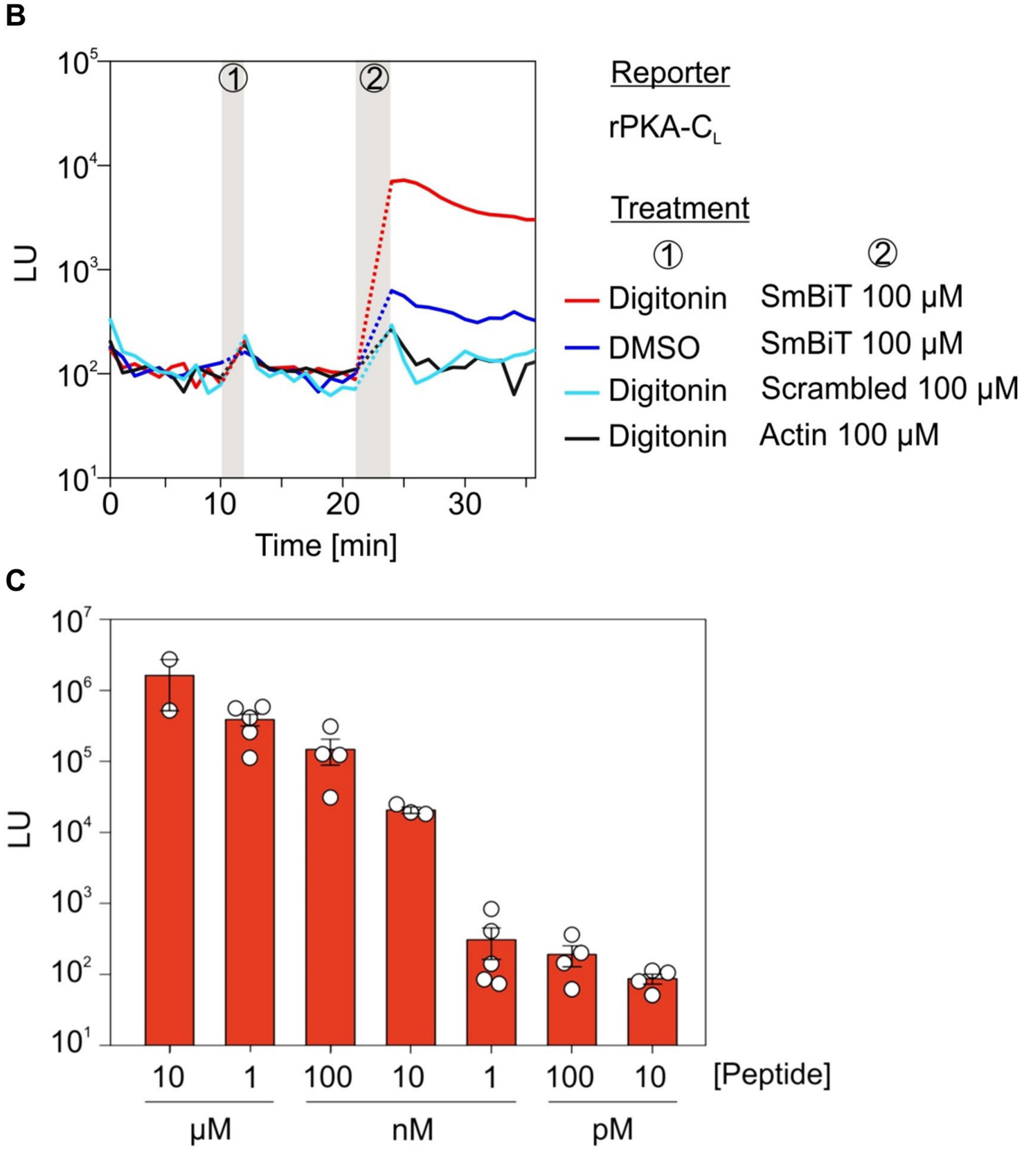

Figure S2. Peptide sequences and membrane permeabilization used for the synthetic complementation assay

(**A**) Peptide sequences and their intended purpose. Positive and negatively charged residues are highlighted in blue and red, respectively. (**B**) 24 h post transfection with rPKA-C_L cells were subjected to the indicated treatment regimens to test effectiveness and necessity of plasma membrane permeabilization for the synthetic peptide complementation. SmBiT or one of the control peptides (scrambled, actin) was supplemented after digitonin or DMSO treatment. Vertical grey bars represent manual application periods without data readings. The dotted lines are extrapolated based on the last and first data points before and after application. (**C**) Additional HaBiT peptide concentrations in micro- and picomolar range were tested in the synthetic complementation assay, but not shown in Fig. 2D. Note that data points for 1, 10, and 100 nM are the same here and in Fig. 2D.

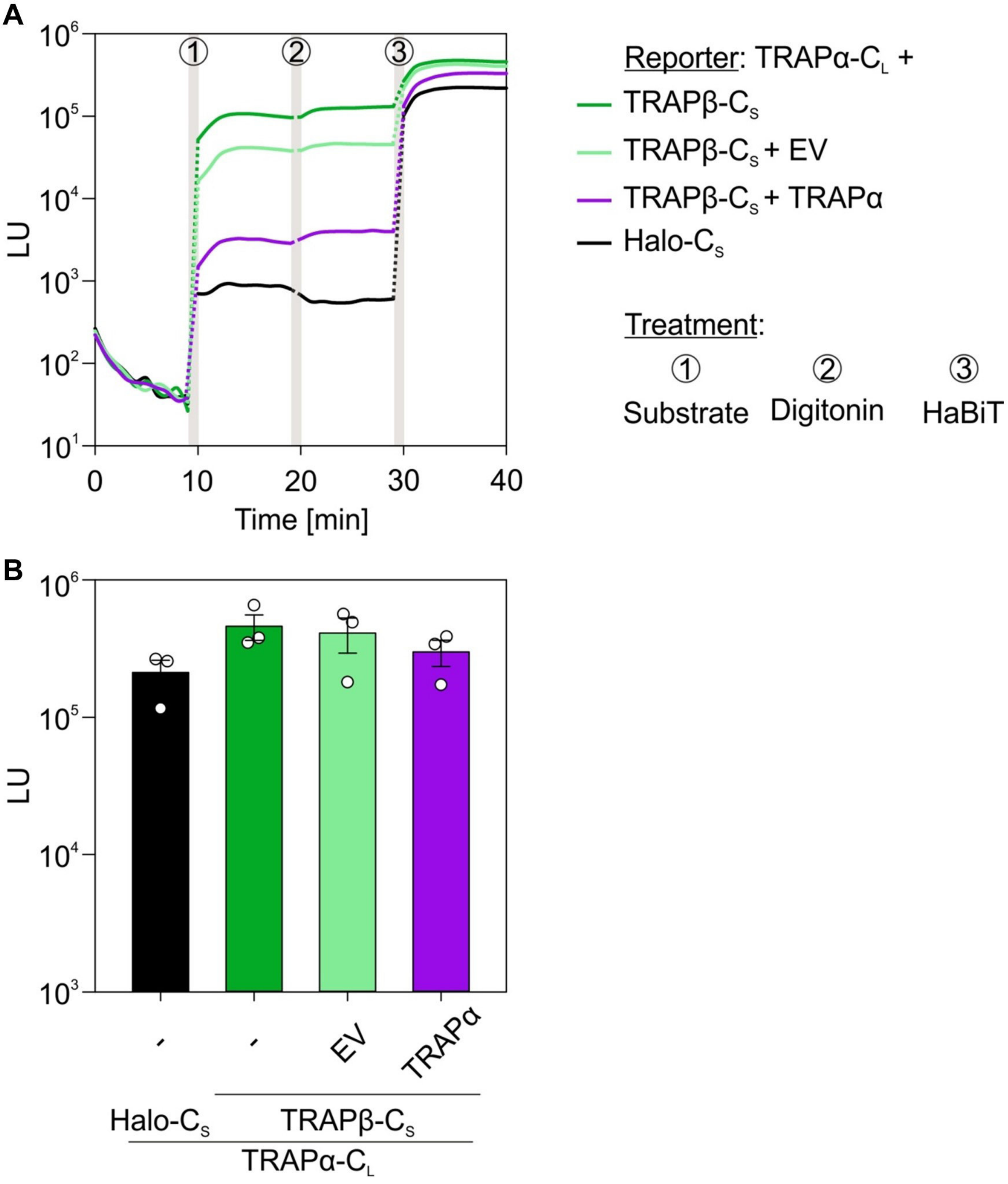

Figure S3. Polytransfections permit efficient and unaltered expression of fusion constructs
(**A**) 24 h after double or triple transfection with the indicated constructs all cells were subjected to the same treatment regimen (circled numbers 1-3) and total luminescent units (LU) were measured. The furimazine treatment to activate luminescence (after 9 min) was followed by permeabilization using digitonin (0.002 %, after 19 min) and peak luminescence was induced by the synthetic HaBiT peptide (100 nM, after 29 min). The high-affinity HaBiT peptide is able to substitute and supersede for SmBiT fusion constructs and was added after digitonin treatment to demonstrate uniform synthesis of TRAPα-C_L irrespective of the number of plasmids mixed for the transfection. A protein name (here TRAPα) without a suffix does not carry a tag and was used to compete with the interaction of the tagged interaction partners. Vertical grey bars represent manual application periods without data readings. The dotted lines are extrapolated based on the last and first data points before and after application. (**B**) Measurements from (**A**) were used for quantification. For each condition and the corresponding biological replicates the signals 4 min after HaBiT addition were evaluated and plotted as LU. Statistical comparison was performed using ANOVA and provided no significant difference for the tested conditions. C_L, C-terminally located LgBiT tag; C_S, C-terminally located SmBiT tag; EV, empty vector

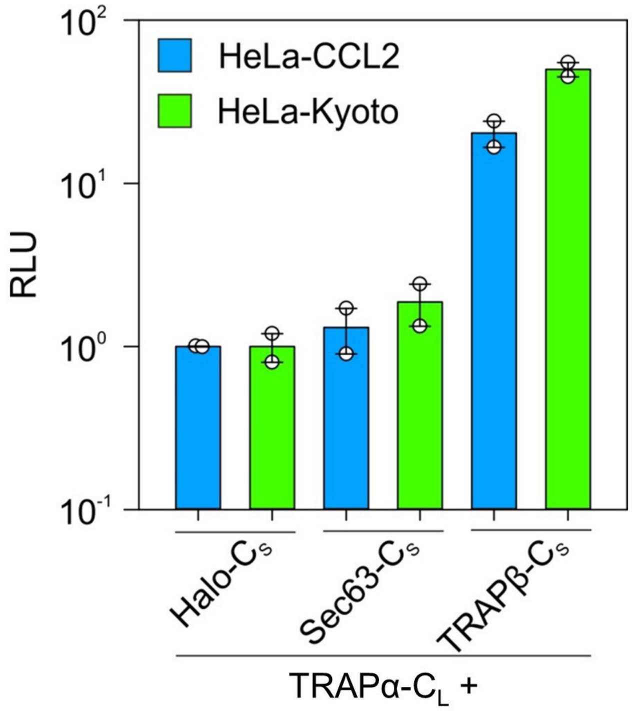

Figure S4. Different cell lines provide analogous results of protein-protein-interactions
24 h post transfection of different cell types (blue and green bars) with the indicated reporter pair the cells were treated with the luminescence activating substrate furimazine. For each condition and the corresponding biological replicates the signals 4 min after furimazine addition were evaluated and plotted as relative luminescent units (RLU). To do so, the luminescence of the negative control pair (TRAPα-C_L plus Halo-C_S) that was run in parallel for each experiment was used for normalization and set to 1. To depict the variation of negative control pair readings the average LU of this condition was used for its normalization in each cell line, respectively. Next to the interacting reporter pair TRAPα-C_L and TRAPβ-C_S the combination of TRAPα-C_L and Sec63-C_S was tested as additional negative control (cf. Fig. 8A, B). In case of the latter combination, physical separation of the LgBiT and SmBiT tags localized in the cytosol and ER lumen prevents BiLC. C_L, C-terminally located LgBiT tag; C_S, C-terminally located SmBiT tag

Table S1. Forward (Fwd) and reverse (rev) PCR primers

Primers were used to amplify the cDNA of interest by PCR and to add the specific cutting sites for restriction enzymes at 5'- and 3'-ends. Sequences matching cDNA are written in uncapitalized letters, overhangs in capital letters, restriction sites in italic and inserted stop codons in bold.

Construct	Fwd primer (5´-3´)	Rev primer (5´-3´)
Sec61α-C_L / Sec61α-C_S	AAGTG*GCTAGC*atggcaatcaaatttc	CCGT*GAATTC*GTgaagagcagggccc
Sec61α-N_L	AGCG*CTCGAG*Gatggcaatcaaatttc	GCC*GCTAGC***TCA**gaagagcagggccc
Sec61β-C_L	TAATACGACTCACTATAGG	CGTC*GAATTC*CTcgaacgagtgtacttgc
Sec61 β-N_S	AGGA*GAATTC*Gatgcctggtccgaccccca	CTC*AGATCT***TTA**cgaacgagtgtacttgc
Sec63-C_S	GCCG*GCTAGC*atggccgggcag	CCG*CTCGAG*CCgtcatcatcttc
Sec63-N_S	CGC*CTCGAG*Catggccgggcag	GCG*GCTAGC***TTA**gtcatcatc
TRAPα-C_L / TRAPα-C_S	TAATACGACTCACTATAGG	GGGCCAC*GAGCTC*Cctcatcagatcccac
TRAPβ-C_L / TRAPβ-C_S	TAATACGACTCACTATAGG	GGGCCAC*GAGCTC*Cgttcttcttcgttttg
